# Long-term low-dose lamotrigine for paroxysmal kinesigenic dyskinesia: a two-year investigation of cognitive function in children

**DOI:** 10.3389/fpsyt.2024.1368289

**Published:** 2024-03-11

**Authors:** Dong-dong You, Yu-mei Huang, Xiao-yu Wang, Wei Li, Feng Li

**Affiliations:** ^1^ Department of Neonatology, The Second Affiliated Hospital and Yuying Children's Hospital of Wenzhou Medical University, Wenzhou, China; ^2^ Department of Pediatric Neurology, The Second Affiliated Hospital and Yuying Children's Hospital of Wenzhou Medical University, Wenzhou, China

**Keywords:** PKD, ADHD, intelligence, PRRT2, follow-up, lamotrigine

## Abstract

**Objective:**

While low-dose lamotrigine has shown effectiveness in managing paroxysmal kinesigenic dyskinesia (PKD) in pediatric populations, the cognitive consequences of extended use are yet to be fully elucidated. This study seeks to assess the evolution of cognitive functions and the amelioration of attention deficit and hyperactivity disorder (ADHD) symptoms following a two-year lamotrigine treatment in children.

**Methods:**

This investigation employed an open-label, uncontrolled trial design. Between January 2008 and December 2021, thirty-one participants, ranging in age from 6.5 to 14.1 years, were enrolled upon receiving a new diagnosis of PKD, as defined by the clinical diagnostic criteria set by Bruno in 2004. Comprehensive evaluation of PRRT2 variants and 16p11.2 microdeletion was achieved using whole-exome sequencing (WES) and bioinformatics analysis of copy number variant (CNV) for all subjects. Immediately after diagnosis, participants commenced treatment with low-dose lamotrigine. Cognitive function was assessed using the Wechsler Intelligence Scale for Children-Chinese Revised (WISC-CR) at baseline and after 2 years, with ADHD diagnoses and symptom severity simultaneously assessed by experts in accordance with the DSM-IV diagnostic criteria for ADHD and the ADHD Rating Scale-IV (ADHD-RS-IV).

**Results:**

Initially, twelve out of 31 patients (38.7%) presented with comorbid ADHD. The latency to treatment initiation was notably longer in PKD patients with ADHD (30.75 ± 12.88 months) than in those without ADHD (11.66 ± 9.08 months), t = 4.856, p<0.001. Notably, patients with a latency exceeding 2 years exhibited a heightened risk for comorbid ADHD (OR = 4.671, P=0.015) in comparison to those with shorter latency. Out of the cohort, twenty-five patients saw the clinical trial to its completion. These individuals demonstrated a marked elevation in WISC-CR scores at the 2-year mark relative to the outset across FSIQ (baseline mean: 108.72 ± 10.45 vs 24 months: 110.56 ± 10.03, p=0.001), VIQ (baseline mean: 109.44 ± 11.15 vs 24 months: 110.80 ± 10.44, p=0.028), and PIQ domains (baseline mean: 106.52 ± 9.74 vs 24 months: 108.24 ± 9.38, p=0.012). Concurrently, a substantial mitigation was observed in ADHD inattention at 2 years compared to baseline (p<0.001), with an average total subscale scores decrement from 9.04 ± 4.99 to 6.24 ± 4.05.

**Conclusion:**

Prolonged duration of untreated PKD in children may elevate the risk of ADHD comorbidity. Notably, following a 2-year lamotrigine regimen, enhancements were observed in both cognitive test outcomes and ADHD symptomatology.

## 1 Introduction

Paroxysmal kinesigenic dyskinesia (PKD; MIM: 128200) falls within a spectrum of movement disorders. It is typified by sporadic episodes of involuntary movements triggered by sudden voluntary actions-especially those involving alterations in speed and amplitude, such as abrupt running or rising from a seated position. Attack frequencies can reach over 100 times daily, with the majority lasting under a minute (1, 2). The proline-rich transmembrane protein 2 (PRRT2) gene emerges as a significant causative factor in PKD, accounting for 77%-93% of familial PKD cases (3). While certain medications effectively manage these episodes, discontinuation frequently results in relapse. Contemporary studies have highlighted potential intellectual and behavioral comorbidities in PKD patients, encompassing developmental delay, intellectual disability, attention deficit hyperactivity disorder (ADHD), anxiety and depression (4–7). However, the evidence remains constrained by small study cohorts and based on PRRT2 gene mutation.

As a commonly used anti-epileptic drug (AED) that appears to be associated with fewer cognitive and behavioral changes than are many other AEDs (8). Existing data suggest that the cognitive deficits are rarely observed in patients receiving Lamotrigine (LTG) monotherapy (9). LTG is as well endorsed as a second-line recommended medication for PKD treatment as per guidelines, yet its influence on cognitive function development in affected children remains understudied. Notably, multiple studies involving epilepsy patients on lamotrigine have indicated an absence of clinically significant cognitive side effects, and even demonstrated enhancements in quality of life (10–12). However, this may be attributed to lamotrigine’s potential in suppressing epileptiform EEG discharges, which could subsequently lead to positive alterations in cognitive function. This study aims to address the critical gap in knowledge by focusing on the effects of long-term low-dose lamotrigine treatment on cognitive functions, specifically ADHD and intelligence, in children with PKD.

## 2 Methods

### 2.1 Study design

This was an open-label, uncontrolled trial in which all PKD participants underwent video-EEG and genetic testing. Evaluations of cognitive function and ADHD symptoms were conducted pre-lamotrigine treatment (baseline) and after 2 years on treatment. The trial protocol encompassed clinical visits for initial screening, baseline assessment, followed by Visits 1-6 at intervals of 1, 2, 6, 12, 18, and 24 months for monitoring.

### 2.2 Patient consent and enrolment

This study was conducted at Department of Pediatric Neurology, The Second Affiliated Hospital and Yuying Children’s Hospital of Wenzhou Medical University. The research adhered to the latest version of the Declaration of Helsinki and secured approval from the institution’s Ethical Committee (2021-K-166-02). Prior to enrollment, written informed consent was procured from the patients or their guardians. The study is registered with the Chinese Clinical Trial Registry (ChiCTR-OPC-15006294).

From January 2008 to December 2021, we enrolled 31 participants, aged between 6.5 and 14.1 years, with newly diagnosed PKD and no biological relationships. All patients were of Han Chinese descent and hailed from Wenzhou city. The inclusion criteria were as follows: 1) No administration of anticonvulsant or ADHD drugs within the past year; 2) Diagnosis of PKD based on Bruno’s 2004 clinical diagnostic criteria (13): (a) Presence of a kinesigenic trigger for the attacks; (b) Short duration of attacks (<1 min); (c) No loss of consciousness or pain during attacks; (d) Exclusion of other organic diseases with normal neurologic examination results; (e) Onset age between 1 and 20 years, in the absence of a family history of PKD.

### 2.3 Treatment of lamotrigine

Upon diagnosis of PKD, all patients were recommended to commence standardized lamotrigine therapy promptly. The lamotrigine dosage was initiated with escalating doses, incrementally adjusted weekly until the attacks were managed. Once effective doses were attained, maintenance doses were administered, confirmed by two consecutive attack-free outpatient visits. Since the commonly used dose of lamotrigine for epilepsy is 1-4mg/kg.d, here, we defined maintenance doses less than 1.5mg/kg.d as low-dose.

### 2.4 Measurements

A video-EEG of over 4 hours was conducted prior to medication initiation. During this procedure, patients were instructed to fall asleep naturally. Upon awakening, patients were prompted to execute their individual trigger actions, such as abrupt movements, to induce an attack. Subsequently, Whole-exome sequencing (WES) and a bioinformatics analysis of copy number variant (CNV) were executed on all patients to analyze PRRT2 variants and the 16p11.2 microdeletion.

Patients’ cognitive abilities were assessed using the Wechsler Intelligence Scale for Children-Chinese Revised Edition (WISC-CR) at both baseline and after 2 years of treatment. The WISC is a recognized and extensively employed intelligence scale tailored for children. The WISC-CR is designed for Chinese children aged between 6 and 16 years, encompassing 12 domains: common sense, analogies, arithmetic, vocabulary, comprehension, digit span, missing picture completion, picture arrangement, block design, object collocation, decoding, and mazes. Scores for each patient were determined based on the operational manual and the respective patient’s age. The first six domains contribute to the verbal intelligence quotient (VIQ), whereas the latter six yield the performance intelligence quotient (PIQ). The cumulative results then allow calculation of the full-scale intelligence quotient (FSIQ) (14).

ADHD evaluations were conducted by investigators following the DSM-IV diagnostic criteria for ADHD at consistent intervals. According to the DSM-IV, ADHD can be categorized into three nominal subtypes based on a specific number of symptoms in two dimensions: inattention and hyperactivity-impulsivity (15). The Predominantly Inattentive Type (ADHD-I) is characterized by individuals exhibiting six or more symptoms of inattention but fewer than six of hyperactivity-impulsivity. The Predominantly Hyperactive-Impulsive Type (ADHD-H) pertains to individuals with six or more hyperactivity-impulsivity symptoms and fewer than six inattention symptoms. Meanwhile, the Combined Type (ADHD-C) encompasses individuals manifesting six or more symptoms across both dimensions (16).

Parent-reports on the ADHD-Rating Scale (ADHD-RS-IV) was administered at baseline and at 2years. It has 18 items covering the symptom criteria for ADHD as outlined in DSM-IV. Parents rated the frequency of each symptom within the past six months on a four-point Likert scale ranging from “Never or rarely” (0), “Sometimes” (1), “Often” (2) to “Very often” (3) with higher scores indicating greater degree of perceived ADHD symptomatology and severity in the child. According to the scale developers, nine items cover deficits in attention and can be summed to generate an Inattention subscale (range: 0–27), whereas the remaining nine items cover hyperactivity and impulsivity and can be summed to yield a Hyperactivity/impulsivity subscale (range: 0–27). A total score can be derived by summing all 18 items (range: 0–54) (17).

### 2.5 Data collection and analysis

Data were analyzed using SPSS software (version 23.0). Alterations in WISC-CR scores and ADHD symptom-items from baseline to the 2-year treatment mark were evaluated with the paired t-test. Group comparisons employed independent samples t-tests and the chi-square test, while simple correlations determined associations between variables. A p-value of < 0.05 was set as the threshold for statistical significance.

## 3 Results

Twenty-one males and 10 females were enrolled, with the mean age 9.6 ± 2.4 years old (range from 6.5 to 14.1 years old). Their mean age of attack onset was 8.0 ± 2.9 years old (range from 3.3 to 13.2 years old) and the mean period before drug administration was 1.6 ± 1.2 years (range from 3 months to 4.1 years).

Of the 31 participants, all exhibited normal results on video-EEGs as well as brain CT or MRI scans. Genetic testing revealed mutations in the PRRT2 gene in 21 patients (67.7%). Three distinct causative heterozygous variants were discerned: the c.649dupC variant (17/21), the c.291delC variant (2/21), and the c.649C>T variant (2/21). Of these, nineteen had a familial history of the disorder, with the criterion being the presence of at least one affected relative within three preceding generations, while 12 emerged as sporadic instances. A notable finding was that five patients had previously encountered afebrile infantile seizures. Parental accounts indicated that attack durations consistently remained under 30 seconds, with frequencies varying from fewer than once daily to in excess of 10 instances per day. In this cohort, 14 patients were sensitive to multiple triggers, while the remaining 17 patients had a singular trigger, namely, sudden movement. The three most prevalent triggers included sudden movement (31/31), intention to move (15/31), and stress (6/31).

Initially, the cognitive abilities of all 31 participants were assessed using WISC-CR. The group’s average FSIQ, VIQ, and PIQ scores were 106.6 ± 11.2, 107.6 ± 11.2, and 104.6 ± 10.5, respectively. None of the participants recorded a FSIQ score below 80. Using the DSM-IV diagnostic criteria, ADHD was diagnosed in 12 participants (38.7%). Meanwhile, ADHD symptoms severity of the participants were assessed according to ADHD-RS-IV. Demographic characteristics of the participants were presented in **Table 1**.

**Table 1:** Demographic and Clinical Characteristics of the PKD Cohort.

Case NO.	sex	Trigger causes	Duration of attacks (sec)	Frequency of attacks (per day)	Family history of paroxysmal dyskinesia	History of afebrile infantile seizure	PRRT2 variants	Age of onset (Y/M)	Illness period before drug therapy (Y/M)	Age (Y/M) of initiating lamotrigine therapy	LTG maintenance dosage (mg/d)	Wt(kg) of initiating lamotrigine therapy	WISC-CR (FSIQ, VIQ, PIQ) at baseline	ADHD symptom-items (I, H) at baseline, ADHD (P/N)	ADHD-RS-IV Total subscale scores (I, H) at baseline	WISC-CR (FSIQ, VIQ, PIQ) at 2 years	ADHD symptom-items (I, H) at 2 years	ADHD-RS-IV Total subscale scores (I, H) at 2 years	Withdrawing during 2 years follow-up
1	M	SM	<15sec	>10 per day	Familial	none	c.649dupC	8Y4M	1Y2M	9Y6M	25	42	88, 95, 84	8, 9, P	22, 21				MP for ADHD
2	M	SM/IM	<20 sec	5–10 per day	AS	none	none	10Y4M	2Y1M	12Y5M	25	48	91, 90, 95	6, 7, P	16, 18				AT for ADHD
3	M	SM	<10 sec	>10 per day	AS	none	c.649dupC	10Y8M	3Y5M	14Y1M	37.5	40	91, 99, 85	6, 8, P	13, 21				MP for ADHD
4	M	SM/IM	<30 sec	5–10 per day	Familial	none	c.649dupC	7Y11M	6M	8Y5M	25	33	120, 126, 110	1, 0, N	4, 3	124, 126, 118	1, 0, N	3, 2	
5	M	SM	<30 sec	1–5 per day	AS	AIS	c.649dupC	6Y3M	3M	6Y6M	25	22	104,100, 107	1, 1, N	6, 5	101, 99, 103	1,1, N	4, 3	
6	M	SM/S	<20 sec	1–5 per day	Familial	none	c.649dupC	5Y0M	2Y3M	7Y3M	12.5	25	98, 105, 91	2,1, N	8, 6	104, 109, 97	1, 1, N	8, 5	
7	F	SM	<10 sec	5–10 per day	Familial	none	c.649dupC	12Y5M	12M	13Y5M	37.5	66	114, 115, 110	7, 5, P	19, 15	115, 115,112	4, 4, N	13, 13	
8	F	SM/IM	<5 sec	1–5 per day	Familial	none	c.291delC	8Y7M	8M	9Y3M	37.5	35	96, 94, 100	0, 0, N	3, 3	99, 94, 105	0, 0, N	1, 2	
9	M	SM/IM	<20 sec	>10 per day	Familial	AIS	c.649dupC	10Y2M	3M	10Y5M	25	44	96, 100, 93	1, 1, N	5, 5	99, 105, 93	0, 1, N	3, 4	
10	F	SM	<30 sec	1–10 per day	Familial	none	c.649dupC	3Y8M	4Y1M	7Y9M	25	35	98, 100, 96	4, 6, P	13, 17	100, 102, 97	4, 4, N	11, 12	
11	F	SM/IM	<30 sec	<1 per day	Familial	none	none	4Y4M	2Y5M	6Y9M	12.5	21	108, 114, 100	3, 2, N	11, 8	111, 116, 103	2, 2, N	7, 8	
12	F	SM	<10 sec	1–10 per day	Familial	none	c.649dupC	11Y3M	10M	12Y1M	25	53	104, 109, 99	2, 1, N	8, 6	106 112, 99	1, 1, N	4, 4	
13	M	SM	<5 sec	1–10 per day	Familial	none	c.291delC	12Y7M	4M	12Y11M	37.5	38	104, 107, 100	4,2, N	14, 6	109, 110, 105	2, 3, N	7, 8	
14	M	SM	<5 sec	1–10 per day	Familial	none	c.649dupC	10Y	1Y7M	11Y7M	25	45	110, 109, 110	7, 6, P	20, 18				AT for ADHD
15	M	SM	<20 sec	1–10 per day	Familial	AIS	c.649dupC	6Y3M	8M	6Y11M	25	18	114, 110, 115	3, 3, N	7, 9	115, 110, 118	1, 1, N	4, 6	
16	M	SM/IM	<30 sec	1–10 per day	Familial	none	c.649dupC	8Y8M	5M	9Y1M	12.5	35	92, 89, 97	2, 0, N	6, 2	95, 92, 99	1, 0, N	4, 2	
17	M	SM/IM/S	<5 sec	<1 per day	AS	none	none	10Y7M	8M	11Y3M	50	60	106, 110, 101	2, 1, N	7, 6				poor compliance to medication
18	F	SM/IM	<20 sec	5–10 per day	AS	none	none	6Y2M	3Y4M	9Y6M	25	38	91, 92, 91	8,6, P	21, 16	95, 99, 92	5, 5, N	15, 13	
19	M	SM	<10 sec	1–10 per day	Familial	AIS	c.649C>T	7Y3M	1Y4M	8Y7M	25	35	100, 96, 104	6, 7, P	17, 20				MP for ADHD
20	F	SM/IM	<20 sec	1–10 per day	AS	none	none	3Y5M	3Y4M	6Y9M	12.5	26	124, 129, 115	3, 6, P	10, 16.	127, 127 120	3, 4, N	9, 11	
21	M	SM/IM/S	<15sec	>10 per day	AS	AIS	c.649dupC	10Y7M	9M	11Y4M	37.5	55	106, 105, 107	1, 1, N	6, 5	109, 110, 107	0, 0, N	3, 2	
22	F	SM/IM	<30 sec	<1 per day	AS	none	none	5Y3M	1Y7M	6Y10M	12.5	24	94, 99, 91	2, 0, N	8, 2	96, 99, 95	1, 1, N	5, 4	
23	M	SM/IM	<20 sec	>10 per day	Familial	none	c.649C>T	13Y3M	6M	13Y9M	37.5	65	119, 120, 115	1, 1, N	6, 7	120, 120, 116	1, 2, N	4, 8	
24	M	SM/S	<20 sec	5–10 per day	AS	none	none	4Y8M	3Y1M	7Y9M	12.5	28	109, 104, 112	4, 8, P	11, 21	111, 105, 115	4, 6, Y	10, 17	
25	M	SM/IM/S	<5 sec	>10 per day	AS	none	none	11Y1M	2Y3M	13Y4M	25	42	109, 105, 112	2, 2, N	8, 7	113, 109., 115	1, 1, N	5, 5	
26	M	SM/IM	<5 sec	5–10 per day	Familial	none	c.649dupC	10Y7M	1Y	11Y7M	25	38	119,120, 114	1, 0, N	5, 1	116, 120, 110	0, 0, N	1, 1	
27	F	SM	<20 sec	1–5 per day	AS	none	none	6Y6M	4M	6Y10M	25	28	114, 112, 114	0, 0, N	1, 2	116, 112, 118	2, 3, N	0, 2	
28	M	SM/IM/S	<50 sec	1–5 per day	Familial	none	c.649dupC	3Y4M	3Y7M	6Y11M	12.5	25	125, 127, 118	5, 6, P	13, 19	128, 130, 120	3, 5, N	9, 16	
29	M	SM	<10sec	5–10 per day	AS	none	none	9Y6M	7M	10Y1M	25	40	116, 112, 118	2, 1, N	8, 7	115, 110, 115	2, 2, N	7, 8	
30	F	SM	<20 sec	1–5 per day	Familial	none	c.649dupC	5Y5M	2Y4M	7Y9M	25	31	120, 117, 119	1, 2, N	7, 6	116, 110, 119	1, 1, N	5, 5	
31	M	SM	<10 sec	>10 per day	Familial	none	c.649dupC	7Y3M	2Y9M	10Y	12.5	36	124, 125., 119	7, 4, P	18, 13	124, 129, 115	5, 2, N	14, 9	

Y, years; M, male/months; F, female; P, positive; N, negative; WISC-CR, Wechsler Intelligence Scale for Children–Chinese revised edition; FSIQ, full-scale intelligence quotient; VIQ, verbal intelligence quotient; PIQ, performance intelligence quotient; ADHD, attention deficit hyperactivity disorder; I, Inattentive Type; H, Hyperactive-Impulsive Type; SM, sudden; movements; IM, intention to move; S, stress; AS, apparently sporadic; AIS, afebrile infantile seizure; MP; Methylphenidate Hydrochloride; AT, Atomoxetine Hydrochloride.

Comparing PKD patients with ADHD to those without, there were no significant differences in trigger causes (p=0.212), duration of attacks (p=0.475), frequency of attacks (p=0.565), PRRT2 variants (p=0.611), family history of paroxysmal dyskinesia (p=0.541), history of afebrile infantile seizure (p=0.342), age of onset (p=0.244), FSIQ score (0.654), VIQ score (0.749), and PIQ score (0.584). The only significant difference between the two groups was that PKD patients with ADHD comorbidity experienced a longer duration before medication initiation (30.75 ± 12.88 months) than those without comorbid ADHD (11.66 ± 9.08 months, t=4.856, p<0.001). Patients waiting over 2 years before initiating therapy exhibited a significantly higher risk for ADHD comorbidity (OR = 4.671, P=0.015) than those with a waiting period less than 2 years. Potential risk factors for ADHD comorbidity in PKD patients can be found in **Table 2**.

**Table 2:** Comparative Analysis of Potential Risk Factors for ADHD Comorbidity in PKD Cohort.

Risk factors		ADHD positive (n= 12), N (%)	ADHD negative (n= 19), N (%)	χ^2/T	P value	OR (95% confidence interval)
Trigger causes	Multiple	5 (41.6)	12 (63.2)	0.641	0.212	0.417 (0.095-1.828)
	Single	7 (58.3)	7 (36.8)			
Duration of attacks (sec)	≥15 sec	6 (50)	11 (57.9)	0.004	0.475	0.727 (0.170-3108)
	<15 sec	6 (50)	8 (42.1)			
Frequency of attacks (per day)	>10 per day	3 (25)	4 (21.1)	0.000	0.565	1.250 (0.226-6.911)
	≤10 per day	9 (75)	15 (78.9)			
PRRT2 variants	positive	8 (66.7)	13 (68.4)	0.000	0.611	0.923 (0.198-4.312)
	negative	4 (33.3)	6 (31.6)			
Family history of paroxysmal dyskinesia	Familial	7 (58.3)	12 (63.2)	0.000	0.541	0.817 (0.186-3.582)
	AS	5 (41.6)	7 (36.8)			
History of afebrile infantile seizure	positive	1 (8.3%)	4 (21.1)	0.191	0.342	0.341 (0.033-3.488)
	negative	11 (91.7)	15 (78.9)			
Age of onset (Years)	≥10	4 (33.3)	8 (42.1)	0.012	0.459	0.688 (0.152-3.102)
	<10	8 (66.7)	11 (57.9)			
Age of onset (Mon)		87.33 ± 37.48	102.26 ± 31.79	-1.1189	0.244	
Illness period before drug therapy (years)	≥2	8 (66.7)	4 (21.1)	4.671	0.015	7.500 (1.469-38.280)
	<2	4 (33.3)	15 (78.9)			
Illness period before drug therapy (Mon)		30.75 ± 12.88	11.66 ± 9.08	4.856	<0.001	
WISC-CR						
	FSIQ	105.42 ± 14.12	107.32 ± 9.29	-0.453	0.654	
	VIQ	106.75 ± 14.01	108.11 ± 9.32	-0.324	0.749	
	PIQ	103.25 ± 12.60	105.42 ± 9.23	-0.554	0.584	

WISC-CR, Wechsler Intelligence Scale for Children–Chinese revised edition; FSIQ, full-scale intelligence quotient; VIQ, verbal intelligence quotient; PIQ, performance intelligence quotient; ADHD, attention deficit hyperactivity disorder.

By the end of the fourth week of lamotrigine therapy, all patients reached an attack-free state. During the follow-up, six patients withdrew from the clinical trial, of whom five took medications for ADHD (cases 1, 2, 3, 14, and 19) and one patient experienced relapses due to poor compliance (case 17). The 25 patients who completed the study underwent reassessment of cognitive ability and ADHD symptoms at the two-year mark (Visit 6).

A notable increase in WISC-CR scores was observed in the cohort of 25 patients following two years of treatment compared to baseline, with significant improvements in FSIQ (mean at baseline 108.72 ± 10.45 vs. at two years 110.56 ± 10.03, p=0.001), VIQ (mean at baseline 109.44 ± 11.15 vs. at two years 110.80 ± 10.44, p=0.028), and PIQ (mean at baseline 106.52 ± 9.74 vs. at two years 108.24 ± 9.38, p=0.012). Concurrently, a pronounced improvement was observed in ADHD inattention symptoms (p<0.001), as reflected by a decline in the mean total subscale scores from 9.04 ± 4.99 to 6.24 ± 4.05. In contrast, ADHD symptoms related to hyperactivity-impulsivity remained statistically unaltered (p=0.328). Moreover, robust correlations were noted between baseline and two-year follow-up assessments in terms of both cognitive scores and ADHD symptoms, specifically in FSIQ (r=0.974, p<0.001), VIQ (r=0.966, p<0.001), PIQ (r=0.946, p<0.001), total inattention subscale scores (r=0.945, p<0.001), and total hyperactivity-impulsivity subscale scores (r=0.955, p<0.001), see **Table 3**. However, We found no statistical differences (all p>0.05) in our study between daily dosage per kilogram and improvements in WISC-CR scores or ADHD symptoms, see **Table 4**.

**Table 3:** Longitudinal Changes in WISC-CR Scores and ADHD Symptomatology Over a 2-Year Treatment Period.

	N	Mean ± SD	Paired comparison t-value	p-value	Correlation r-value	p-value
FSIQ at baseline	25	108.72 ± 10.45	-3.738	0.001	0.974	<0.001
FSIQ at 2 years	25	110.56 ± 10.03				
VIQ at baseline	25	109.44 ± 11.15	-2.334	0.028	0.966	<0.001
VIQ at 2 years	25	110.80 ± 10.44				
PIQ at baseline	25	106.52 ± 9.74	-2.714	0.012	0.946	<0.001
PIQ at 2 years	25	108.24 ± 9.38				
Total subscale scores (I) at baseline	25	9.04 ± 4.99	2.178	0.034	0.945	<0.001
Total subscale scores (I) at 2 years	25	6.24 ± 4.05				
Total subscale scores (H) at baseline	25	8.28 ± 5.86	0.989	0.328	0.955	<0.001
Total subscale scores (H) at 2 years	25	6.80 ± 4.65				

WISC-CR, Wechsler Intelligence Scale for Children–Chinese revised edition; ADHD, attention deficit hyperactivity disorder; FSIQ, full-scale intelligence quotient; VIQ, verbal intelligence quotient; PIQ, performance intelligence quotient; Total subscale scores (I),Total inattention subscale score; Total subscale scores (H), Total hyperactivity/impulsivity subscale score.

**Table 4:** Relationship between dosage per kilogram and improvements in WISC-CR Scores or ADHD Symptomatology.

Number	4	5	6	7	8	9	10	11	12	13	15	16	18	20	21	22	23	24	25	26	27	28	29	30	31	r	P
LTG maintenance dosage (mg/d.kg)	0.38	0.28	0.25	0.38	1.07	0.85	0.36	1.19	0.47	0.66	1.39	1.43	0.99	0.48	0.45	2.08	0.38	0.89	1.19	0.66	0.45	0.50	0.94	0.81	1.39		
△FSIQ	4	-3	6	1	3	3	2	3	2	5	1	3	4	3	3	2	1	2	4	-3	2	3	-1	-4	0	-0.002	0.993
△VIQ	0	-1	4	0	0	5	2	2	3	3	0	3	7	-2	5	0	0	1	4	0	0	3	-2	-7	4	0.064	0.763
△PIQ	8	-4	6	2	5	0	1	3	0	5	3	2	1	5	0	4	1	3	3	-4	4	2	-3	0	-4	-0.002	0.993
△subscale scores (I)	-1	-2	0	-6	-2	-2	-2	-4	-4	-7	-3	-2	-6	-1	-3	-3	-2	-1	-3	-4	-1	-4	-1	-2	-4	0.064	0.763
△subscale scores (H)	-1	-2	-1	-2	-1	-1	-5	0	-2	2	-3	0	-3	-5	-3	2	1	-4	-2	0	0	-3	1	-1	-4	-0.002	0.993

WISC-CR, Wechsler Intelligence Scale for Children–Chinese revised edition; ADHD, attention deficit hyperactivity disorder; △FSIQ, full-scale intelligence quotient at 2 years - full-scale intelligence quotient at baseline; △VIQ, verbal intelligence quotient at 2 years - verbal intelligence quotient at baseline; △PIQ, performance intelligence quotient at 2 years - performance intelligence quotient at baseline; △subscale scores (I), Total inattention subscale score at 2 years - Total inattention subscale score at baseline; △subscale scores (H), Total hyperactivity/impulsivity subscale score at 2 years - Total hyperactivity/impulsivity subscale score at baseline.

## 4 Discussion

Paroxysmal kinesigenic dyskinesia (PKD) is the most common form of paroxysmal dyskinesia. Typically triggered by sudden voluntary movements, PKD manifests as brief episodes of movement disorders such as bilateral chorea, ballism, or dystonia, with patients maintaining consciousness during these episodes (18, 19). Over 40% of patients are initially misdiagnosed upon their first medical consultation. Patients often spend years, sometimes decades, seeking an accurate diagnosis and appropriate treatment (20). In addition there is still a very lack of research on cognitive function in children with PKD patients, only very few studies found individuals carrying either homozygous or compound-heterozygous present with a higher risk of intellectual disability and learning difficulties than those with heterozygous PRRT2 mutations (6, 21). This suggests that PRRT2 mutations have a gene-dosage effect and that other factors critically influence PRRT2-PKD symptoms.

Approximately more than half of global PKD cases are linked to PRRT2 mutations, underscoring their close association (22–25). Although not much is known about the PRRT2 protein, it is known to interact with SNAP25, a pre-synaptic membrane protein, which has led to the theory that the disease pathogenesis may be related to synaptic dysfunction (26, 27). In addition, PRRT2 mutations are hypothesized to interfere with the glutamatergic signal, leading to neuronal hyperexcitability by glutamate (28). Findings from animal model studies of PKD support the theory that hyperexcitability in the basal ganglia underlies these symptoms (29, 30). Most studies indicate that γ-aminobutyric acid acts as the principal inhibitory neurotransmitter in the basal ganglia, dominating information processing across its various regions (20, 31). A recent study highlighted that PRRT2 mutations might directly impact synaptic vesicle cycling (32–35). Notably, this cycling process is associated with a spectrum of neurological symptoms such as movement disorders and epilepsies, cognitive impairments including developmental delay and intellectual disabilities, as well as mental health challenges like autism and ADHD (4, 36, 37).

ADHD is the most common neurobehavioral disorder in children, marked by inattention, impulse and anger control problems, influenced by genetic and environmental factors. The worldwide-pooled prevalence of ADHD is 5.29% (38). The mechanisms underlying ADHD are still unclear, monoamine neurotransmitters are still most valued (39, 40), but an imbalance of glutamic acid/γ-aminobutyric acid may also be present (41). As is mentioned above, glutamate is the major excitatory neurotransmitter, meanwhile, γ-aminobutyric acid is the major inhibitory neurotransmitter in the brain. A study has demonstrated hyperactivity can be produced by pharmacologically increasing the activity of the nucleus accumbens core using glutamatergic (42). And another study has showed children with ADHD had lower γ-aminobutyric acid levels in a region including parts of the primary somatosensory and motor cortices (43).

To the best of our understanding, limited studies have been conducted on the outcomes of cognitive functions and behavioral impairments in children with PKD, both in China and internationally. An earlier study with a limited sample size revealed that psychiatric comorbidities such as obsessive-compulsive disorder, depression, Autism spectrum disorder, tic disorder, and attention deficit hyperactivity disorder (ADHD) were observed in 6/14 patients with PKD (42.9%) (44). A cross-sectional survey of 165 primary PKD patients highlighted that depression, anxiety, and low levels of quality of life were prevalent among these patients (45, 46). Certain research suggests that the PRRT2 loss of function in the hippocampus and frontal lobe of the cerebral cortex might induce neuronal hyperexcitability and synaptic deregulation, leading to compromised cognitive performance (47).

Our findings align with previous studies (37), highlighting a higher prevalence of ADHD (12/31) in children with PKD compared to the general population (5.32%). However, we noted that the average cognitive function in PKD patients did not deviate from the normal range, and PRRT2 variants did not contribute to the prevalence of ADHD. We hypothesize that genetic modifiers, age-dependent transcriptional profiles, or distinct environmental factors might influence the condition of neurodevelopment. Interestingly, the only significant predictor for comorbid ADHD in patients with PKD was a relatively longer period before drug therapy, indicating that it is potentially a consequence of psychosocial disadvantages associated with enduring PKD symptoms or the cumulative negative impacts of living with the illness. We posit that attacks in PKD are more likely a dysfunction of the basal ganglia, which do not lead to neurobiological consequences of plasticity or apoptosis. Nonetheless, the causal relationship among ADHD, FSIQ, and PKD remains inconclusive.

Moreover, our study revealed that patients who had never received specialized treatment for ADHD experienced significant alleviation of ADHD symptoms after two years of consistent medication for PKD. They also achieved significantly higher scores on FSIQ and VIQ. This significant alleviation of ADHD symptoms might lead to improved academic achievement and reduce the risk of several negative outcomes. The practical implications of minor improvements in IQ scores remain uncertain. Still, a plausible explanation could be that the medication reduced disturbances from both PKD and ADHD symptoms, thereby enabling the children to tap into more of their cognitive potential.

The effect of lamotrigine on cognition remains a topic of debate (10). Several studies have indicated that lamotrigine is not associated with impaired cognitive function and is effective in treating bipolar disorder (48). Moreover, it may enhance cognitive function and health-related quality of life (9, 49). Conversely, other studies have suggested that long-term lamotrigine treatment might lead to types of cognitive impairments not observed during short-term treatment (10). Nevertheless, no conclusive evidence exists that lamotrigine therapy directly affects cognitive function or ADHD symptoms.

Our study indicates Prolonged duration of untreated PKD in children may elevate the risk of ADHD comorbidity and provides evidence of significant cognitive improvement and ADHD symptom mitigation in children with PKD following a two-year treatment with low-dose lamotrigine. These findings recommended clinicians to screen all children with PKD for ADHD-related symptoms, meanwhile, a proactive and comprehensive treatment s is probably necessary.

Our study had several limitations, including the open uncontrolled design, which suggests that factors other than the treatment of lamotrigine might have influenced the results, and the relatively small sample size. Despite neither the patients nor their parents reporting specific ADHD symptoms, neuropsychological tests were conducted as screening tests for all newly diagnosed PKD patients. This might have led parents to respond more positively on the questionnaires.

## Data availability statement

The original contributions presented in the study are included in the article/**Supplementary Material**. Further inquiries can be directed to the corresponding authors.

## Ethics statement

The studies involving humans were approved by The Second Affiliated Hospital and Yuying Children’s Hospital of Wenzhou Medical University. The studies were conducted in accordance with the local legislation and institutional requirements. Written informed consent for participation was not required from the participants or the participants’ legal guardians/next of kin in accordance with the national legislation and institutional requirements. Written informed consent was obtained from the minor(s)’ legal guardian/next of kin for the publication of any potentially identifiable images or data included in this article.

## Author contributions

FL: Resources, Writing – original draft. D-DY: Data curation,Investigation, Writing–original draft. Y-MH: Data curation,Funding acquisition, Methodology, Resources, Writing–original draft, Writing – review & editing. X-YW: Data curation, Investigation, Methodology, Writing – review & editing. WL: Data curation, Writing – original draft, Writing – review & editing.
